# Tissue biodistribution and tumor targeting of near-infrared labelled anti-CD38 antibody-drug conjugate in preclinical multiple myeloma

**DOI:** 10.18632/oncotarget.28074

**Published:** 2021-09-28

**Authors:** Nicholas Cho, Sooah Ko, Monica Shokeen

**Affiliations:** ^1^Department of Radiology, Washington University School of Medicine, St. Louis, MO, USA; ^2^Department of Biomedical Engineering, Washington University in St. Louis, St. Louis, MO, USA; ^3^Alvin J. Siteman Cancer Center, Washington University School of Medicine and Barnes-Jewish Hospital, St. Louis, MO, USA

**Keywords:** near-infrared (NIR) fluorescence, multiple myeloma (MM), cluster of differentiation 38 (CD38), antibody-drug conjugate (ADC), small animal optical imaging

## Abstract

Daratumumab (DARA) is an FDA-approved high-affinity monoclonal antibody targeting CD38 that has shown promising therapeutic efficacy in double refractory multiple myeloma (MM) patients. Despite the well-established clinical efficacy of DARA, not all heavily pretreated patients respond to single-agent DARA, and the majority of patients who initially respond eventually progress. Antibody-drug conjugates (ADCs) combine the highly targeted tumor antigen recognition of antibodies with the cell killing properties of chemotherapy for effective internalization and processing of the drug. In this study, we evaluated the anti-tumor efficacy of DARA conjugated to the maytansine derivative, mertansine (DM1), linked via a non-cleavable bifunctional linker. The ADC was labelled with the near-infrared (NIR) fluorophore IRDye800 (DARA-DM1-IR) to evaluate its stability, biodistribution and pharmacokinetics *in vitro* and *in vivo*. We demonstrated the conjugation of: 1) DM1 enhanced tumor-killing efficacy of the native DARA and 2) IRDye800 allowed for visualization of uptake and tumor targeting ability of the ADC. With the advent of other classes of immunoconjugates for use in MM, we reasoned that such imaging techniques can be utilized to evaluate other promising conjugates in preclinical MM models on a whole-body and cellular level.

## INTRODUCTION

Daratumumab (DARA) is a human IgG1 monoclonal antibody that targets Cluster of Differentiation 38 (CD38), inducing tumor cell death through multiple mechanisms, including antibody-dependent cell-mediated cytotoxicity (ADCC), complement-dependent cytotoxicity, and antibody-dependent cellular phagocytosis (ADCP) [1]. Despite the well-established clinical efficacy of DARA in relapsed and refractory multiple myeloma (MM) patients, not all of the heavily pretreated patients respond to single-agent DARA therapy, and the majority of patients who initially respond eventually progress [2]. This may be due to upregulation of pathways that inhibit DARA-mediated ADCC and CDC [3]. One possibility for enhancing the potency of DARA and increasing its therapeutic index is to introduce additional drugs to complement the multiple mechanisms of action of the native immunotherapy. Antibody-drug conjugates (ADCs) combine the highly targeted tumor antigen recognition of antibodies with the cell killing properties of chemotherapy for effective internalization and processing of the drug. The ADC is designed to provide a wider therapeutic window than the antibody alone or the parent cytotoxic drug payload attached to it. Drugs such as maytansines are potent tubulin inhibitors that have previously failed FDA approval due to their poor therapeutic window and lack of tumor specificity, but have demonstrated excellent stability and acceptable solubility in aqueous solutions for use in other clinically-approved ADCs [4]. In this study, we evaluated the anti-tumor efficacy of DARA conjugated to the maytansine derivative, mertansine (DM1), linked *via* the non-cleavable bifunctional linker succinimidyl 4-(N-maleimidomethyl)cyclohexane-1-carboxylate (SMCC) (DARA-DM1). The SMCC linker contains a thioether bond, requiring complete lysosomal degradation of the ADC for intracellular release of the payload, and has demonstrated improved *in vivo* stability and reduced off-target toxicity compared to ADCs with cleavable linkers [5]. The cleaved drug product, lysine-SMCC-DM1, contains a net positive charge, allowing for improved retention in the target cell following internalization of the ADC [6]. We posit that DARA conjugated to DM1 *via* a non-cleavable linker will enhance the potency of the native DARA while maintaining high MM tumor specificity and *in vivo* stability.

Binding and internalization of an ADC play critical roles in a biologic’s overall therapeutic potential and delivery of the cytotoxic drug to the target tumor cell. Molecular imaging can serve as a powerful tool to evaluate uptake of antibody-based therapies and provide significant insights into designing next-generation therapeutic agents with superior safety and efficacy. Traditionally, such imaging studies are performed through imaging of radiolabeled compounds with Positron Emission Tomography (PET) or Single-Photon Emission Computed Tomography (SPECT). While PET and SPECT are highly sensitive and can be used to measure tracer uptake into tissues, the use of decaying radioisotopes and ability to trace just one molecular species (in the case of PET) in a given imaging experiment does not allow for longitudinal monitoring of interactions between molecular targets [7]. Labelling with fluorescent probes for optical imaging in the first near-infrared (NIR-I) window (650–950 nm) allows for reduced autofluorescence *in vivo* than in the visible fluorescence range (400–650 nm) on a whole-body and cellular level in preclinical animal models [8]. While there have been preclinical efforts in developing fluorescently-labelled ADCs for solid cancers [9, 10], there have been no published studies evaluating fluorescently-labelled ADCs in preclinical models of MM or other hematologic malignancies.

Here, we labelled DARA-DM1 with the NIR fluorophore IRDye800 (Ex./Em. 774 nm/810 nm) (DARA-DM1-IR). In addition to efficacy, we evaluated specificity of the ADC, in relation to the native antibody, to myeloma tumor cells both *in vitro* and *in vivo*. We hypothesize that: 1) the conjugation of the drug will enhance the therapeutic efficacy of DARA without affecting tumor targeting of the DARA antibody and 2) labelling with the NIR fluorophore will allow for visualization of DARA-DM1 on a whole-body and cellular level. The proof of principle studies in this article demonstrated the potential of NIR fluorescence imaging for evaluating the cellular uptake and biodistribution of antibody-based therapies in preclinical MM and other hematologic cancers.

## RESULTS

### Synthesis and characterization of DARA-DM1 and DARA-DM1-IR

DM1 was conjugated to DARA at a molar ratio of 20 to 1. Mass spectrometry was performed on DARA-DM1 to calculate a drug to antibody ratio (DAR) of 3.2 with less than 10% of unconjugated DARA remaining following DM1 conjugation (Supplementary Figure 1A). Following DM1 conjugation, IRDye800 was labelled to DARA-DM1 and DARA at a dye to antibody ratio of 3 to 1. Absorption spectroscopy showed a similar degree of labelling (DOL) of IRDye800 to both DARA-DM1 and DARA at ~1.3–1.4 (Supplementary Figure 1B). Fluorescence spectroscopy confirmed that there was not a difference in brightness between the two conjugates (Supplementary Figure 1C).

### Cytotoxicity of DARA-DM1 and DARA-DM1-IR

**Figure 1:**
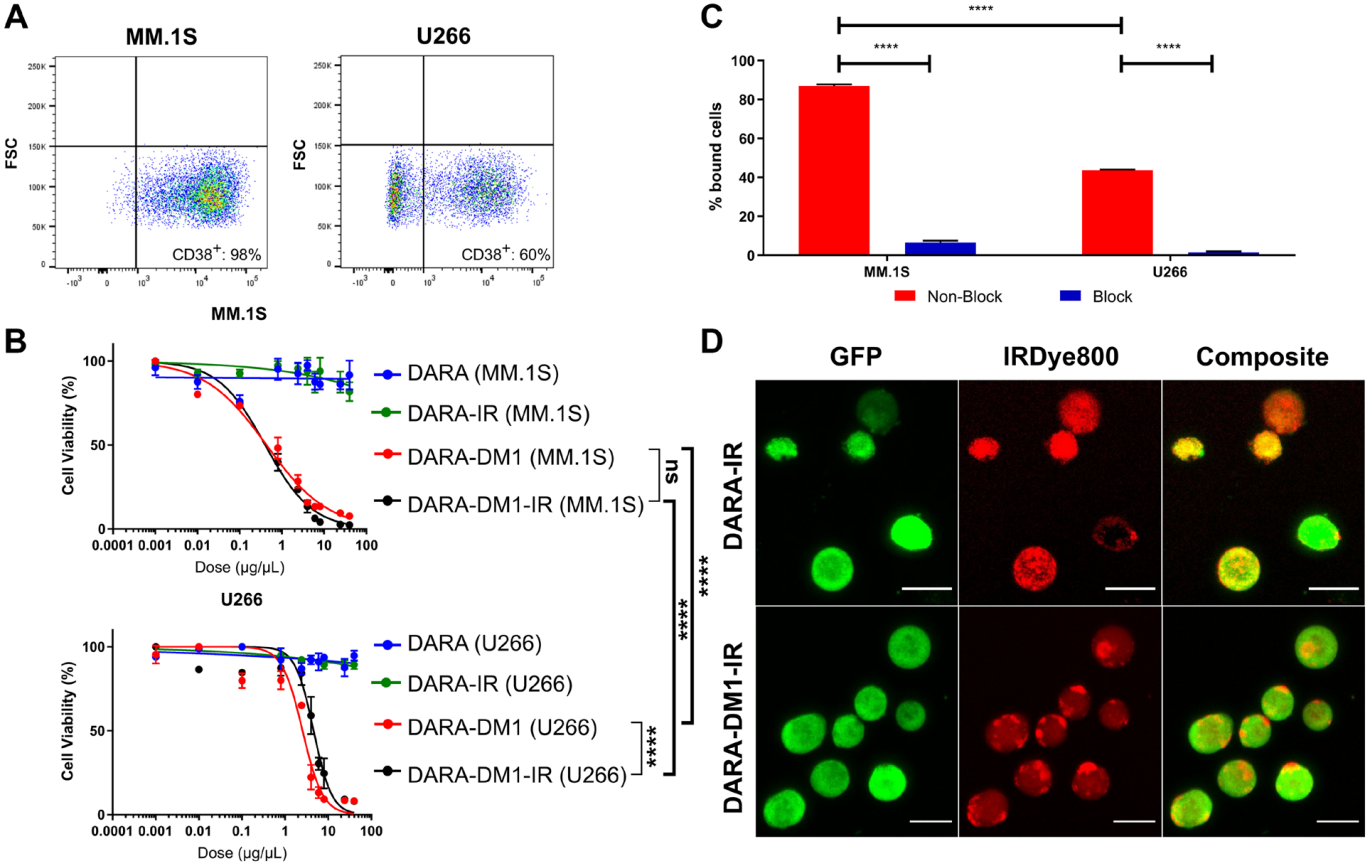
*In vitro* evaluation of cytotoxicity, binding and internalization of DARA-DM1-IR. (**A**) Flow cytometric evaluation of CD38 expression in human MM.1S and U266 myeloma cells. (**B**) Comparison of cytotoxicity of DARA-DM1 and DARA-DM1-IR and control DARA and DARA-IR in human MM.1S and U266 myeloma cells. All experiments were performed in triplicate and repeated twice. Two-way ANOVA followed by Sidak’s multiple comparison’s test was performed on IC_50_ values. (**C**) Percentage cell binding of DARA-DM1-IR in MM.1S and U266 cells at 37°C in the absence and presence of 50-fold blocking dose of unlabelled DARA. Two-way ANOVA followed by Sidak’s multiple comparison’s test was performed. (**D**) Live cell fluorescence microscopy of internalization of DARA-IR and DARA-DM1-IR in MM.1S-GFP-luc cells 3 hours post-incubation. Magnification: 20×; Scale bar: 20 μm. ^****^ *p* < 0.0001. Error bars represent standard deviation.

To evaluate the *in vitro* cytotoxicity of DARA-DM1 compared to the native DARA antibody, DARA conjugates were incubated with two human myeloma cell lines, MM.1S and U266, in a dose-dependent fashion. CD38 expression was evaluated on both cell lines *via* flow cytometry showing high and low expression of CD38 on MM.1S and U266 cell lines, respectively (Figure 1A). DARA-DM1 and DARA-DM1-IR both exhibited a statistically significant difference in cytotoxicity in MM.1S cells (DARA-DM1 IC_50_: 0.43 ± 0.05 μg/mL; DARA-DM1-IR IC_50_: 0.40 ± 0.03 μg/mL) compared to U266 cells (DARA-DM1 IC_50_: 2.54 ± 0.4 μg/mL; DARA-DM1-IR IC_50_: 4.58 ± 0.7 μg/mL) (*p* < 0.0001). Conjugation of IRDye800 to DARA-DM1 did not show a statistical difference in tumor-killing ability when compared to DARA-DM1 in MM.1S cells, but showed a statistical difference in U266 cells (*p* < 0.0001) (Figure 1B).

### Evaluation of stability, binding and internalization properties of IRDye800-conjugated antibodies

Stability of DARA-IR and DARA-DM1-IR in Phosphate Buffer Saline (PBS) and human serum was measured over 7 days. Both IRDye800 conjugates maintained >80% NIR signal by day 7, indicating minimal dye deconjugation and formation of free dye during incubation (Supplementary Figure 2). Flow cytometry and fluorescence microscopy were performed to determine if conjugation of IRDye800 to the ADC perturbed the antibody’s binding property. DARA-DM1-IR demonstrated significant binding in MM.1S and U266 cells in a CD38-dependent manner (MM.1S: 86.8 ± 0.9%; U266: 43.6 ± 0.4%). Blocking of CD38 receptor with excess DARA demonstrated significantly reduced binding of DARA-DM1-IR in both cell lines (MM.1S: 6.46 ± 1.1%; U266: 1.53 ± 0.5%) (Figure 1C). Evaluation by flow cytometry of the lysosomal-associated membrane protein 1 (LAMP-1) staining in MM.1S and U266 cells corresponded with binding, showing significant intracellular internalization of both DARA conjugates in MM.1S cells (DARA-IR: 96.2 ± 0.3%; DARA-DM1-IR: 98.5 ± 0.1%), but significantly reduced internalization in U266 cells (DARA-IR: 12.7 ± 0.7%; DARA-DM1-IR: 12.2 ± 1.8%) (Supplementary Figure 3). Fluorescence microscopy of MM.1S cells incubated with both DARA conjugates confirmed flow cytometric LAMP-1 staining results, showing significant internalization within 3 hours (Figure 1D).

### *In vivo* therapeutic efficacy of DARA-DM1

**Figure 2:**
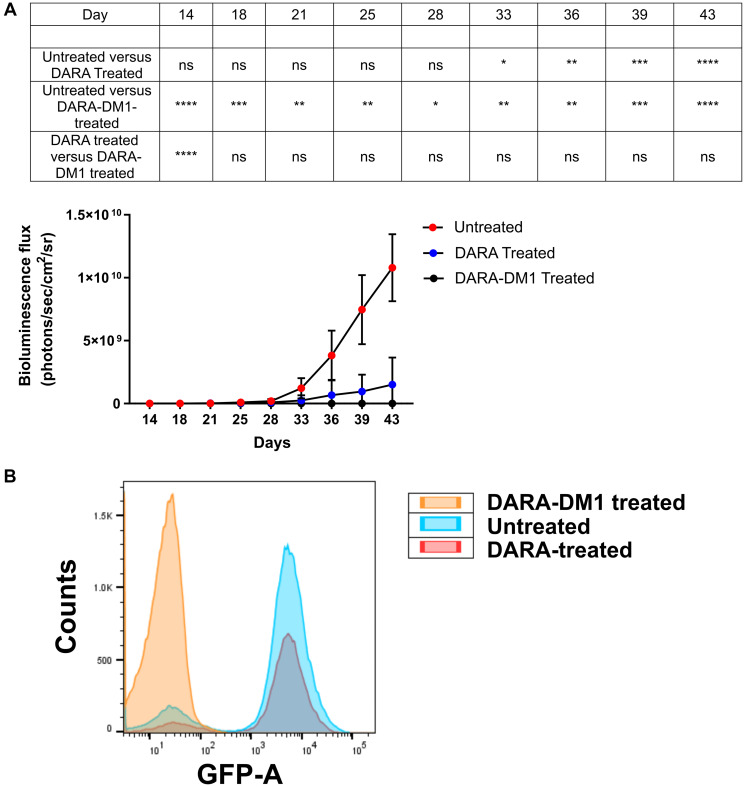
*In vivo* therapeutic efficacy of DARA-DM1 in intramedullar myeloma mice. (**A**) Longitudinal BLI of MM.1S IV mice treated with a single dose of DARA or DARA-DM1 measured as bioluminescence flux (photons/sec/cm^2^/sr). *n* = 6–7/group. Repeated measures two-way ANOVA followed by Sidak’s multiple comparison’s test was performed. (**B**) Representative flow cytometry from excised bone marrow of untreated and treated mice, gating for live, GFP^+^ MM cells. ^*^ *p* < 0.05; ^**^ *p* < 0.01; ^***^ *p* < 0.001; ^****^ *p* < 0.0001. Error bars represent standard deviation.

To demonstrate the therapeutic efficacy of DARA-DM1 *in vivo*, fox chase severe combined immunodeficient (SCID) beige mice injected intravenously (IV) with MM.1S cells transfected with green fluorescence protein (GFP) and luciferase (MM.1S IV) were treated with either DARA or DARA-DM1 at doses of 4 mg/kg of body weight and were monitored with bioluminescence imaging (BLI). DARA-DM1 resulted in significant tumor eradication following single administration and showed sustained reduction in tumor burden at all BLI time points when compared to untreated mice. Unconjugated DARA, conversely, began demonstrating a significant reduction in tumor burden only at 33 days following tumor inoculation (Figure 2A). A significant difference in tumor burden was also observed in mice treated with DARA-DM1 compared to DARA at Day 14 (*p* < 0.0001). No significant weight loss was demonstrated in any of the untreated or treated (DARA and DARA-DM1) mice (Supplementary Figure 4). *Ex vivo* flow cytometry on excised femoral and pelvic bone marrow, gated for live, GFP^+^ tumor cells, verified the anti-MM effect seen with *in vivo* BLI results (Figure 2B).

### High specificity of DARA-DM1-IR conjugate to CD38^+^ myeloma extramedullary tumors

**Figure 3:**
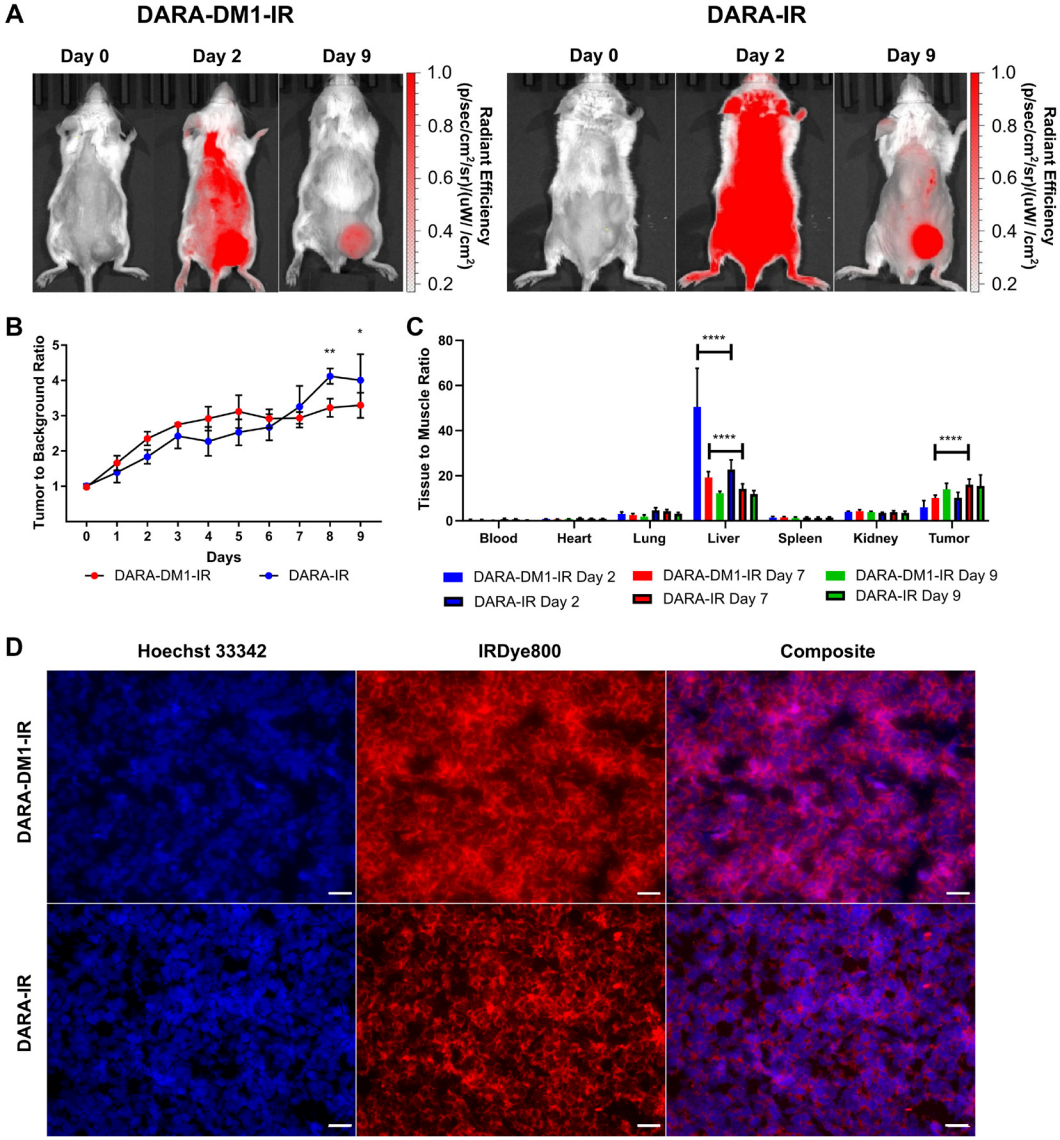
High contrast observed with DARA-DM1-IR at longer time points in MM.1S SQ mice. (**A**) Representative IRDye800 *in vivo* images of MM.1S SQ mice 2 and 9 days post administration of DARA-DM1-IR and DARA-IR. (**B**) Plot of calculated Tumor to Background Ratios (TBRs) in MM.1S SQ mice across individual time points following administration of DARA-DM1-IR and DARA-IR. Background is defined as the non-tumor, contralateral left flank of the mouse. Repeated measures two-way ANOVA followed by Sidak’s multiple comparison’s test was performed on TBR data. *n* = 3–4/group. (**C**) Normalized biodistribution (defined as tissue to muscle ratio (TMR)) of DARA-DM1-IR 2, 7 and 9 days after administration of fluorescent conjugate. *n* = 3–4/group. Two-way ANOVA followed by Sidak’s multiple comparisons test was performed on biodistribution data. (**D**) Immunohistochemistry of excised tumor sections from mice injected with DARA-DM1-IR and DARA-IR. Nuclear stain was performed with Hoechst 33342. Magnification: 40×; Scale bar: 100 μm. ^*^ *p* < 0.05; ^**^ *p* < 0.01; ^****^ *p* < 0.0001. Error bars represent standard deviation.

To quantify contrast and the optimal imaging time point of DARA-DM1-IR, SCID beige mice bearing subcutaneous (SQ) tumor xenografts (MM.1S SQ) were injected IV with the fluorescent conjugate. Significant uptake of DARA-DM1-IR was observed in GFP^+^ tumor-bearing regions by day 9 of fluorescent imaging (Figure 3A). Region of Interest (ROI) analysis of small-animal fluorescent imaging with DARA-DM1-IR showed high contrast, calculated as Tumor to Background Ratio (TBR), at later time points, reaching a peak of 3.3 ± 0.4 at day 9 as compared to a TBR of 4.0 ± 0.7 in mice injected with DARA-IR (Figure 3B). This optimal time point informed the imaging in studies involving the MM.1S IV mouse model. A statistically significant difference in TBRs between DARA-DM1-IR and DARA-IR was observed at Day 8 (*p* < 0.01) and 9 (*p* < 0.05). Tissue biodistribution studies performed in the IRDye800 channels at 2, 7 and 9 days post administration of DARA-DM1-IR and DARA-IR were in agreement with the *in vivo* fluorescent imaging data, calculated as Tissue to Muscle Ratio (TMR), showing high uptake and retention of the fluorescent conjugate 9 days after injection (DARA-DM1-IR TMR: 13.9 ± 2.6; DARA-IR TMR: 15.5 ± 4.8) (Figure 3C). Significant differences in non-tumor tissue were observed primarily in the liver with greater uptake of DARA-DM1-IR at day 2 (DARA-DM1-IR TMR: 50.6 ± 17.1; DARA-IR TMR: 22.8 ± 4.2) and day 7 (DARA-DM1-IR TMR: 19.2 ± 2.6; DARA-IR TMR: 14.2 ± 2.2) (Figure 3C). Similar liver uptake was observed between the two antibody conjugates by day 9 (DARA-DM1-IR TMR: 12.2 ± 0.9; DARA-IR TMR: 11.9 ± 1.6). Immunofluorescence staining of excised tumors supported *in vivo* and *ex vivo* imaging results and demonstrated specific binding of DARA-DM1-IR, similarly to DARA-IR, to tumor cells (Figure 3D).

### High specificity of DARA-DM1-IR conjugate to CD38^+^ myeloma intramedullary tumors

**Figure 4:**
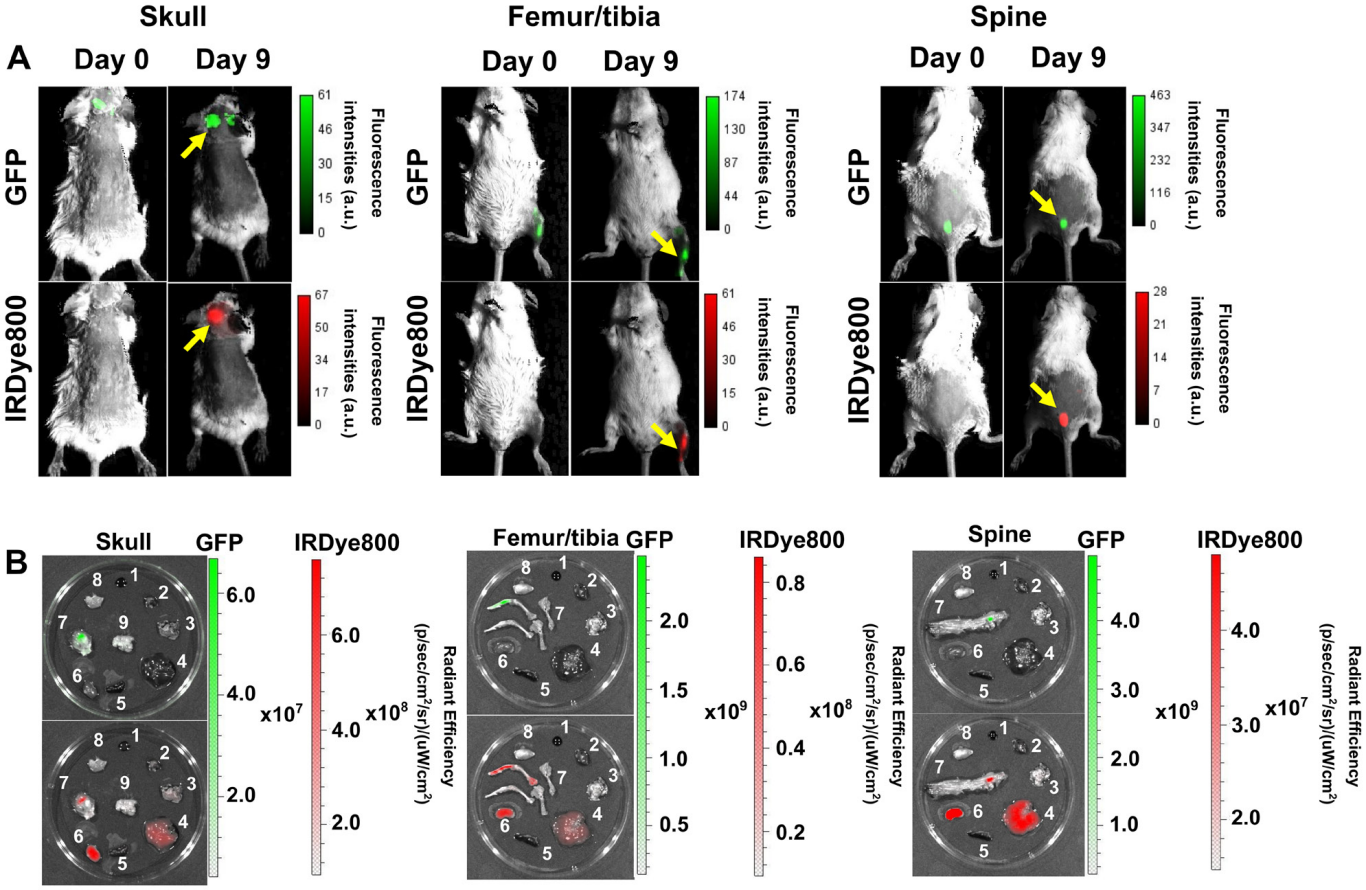
Specific binding of DARA-DM1-IR to cancerous bone marrow regions in MM.1S IV mice. (**A**) Representative images of GFP and IRDye800 fluorescence *in vivo* of skull, long bones and spine (yellow arrows) in separate MM.1S IV models 9 days post administration of DARA-DM1-IR. (**B**) Representative GFP and IRDye800 fluorescent images of excised 1) blood 2) heart 3) lung 4) liver 5) spleen 6) kidney 7) bone 8) muscle 9) brain from mice. a.u. stands for arbitrary units.

**Figure 5:**
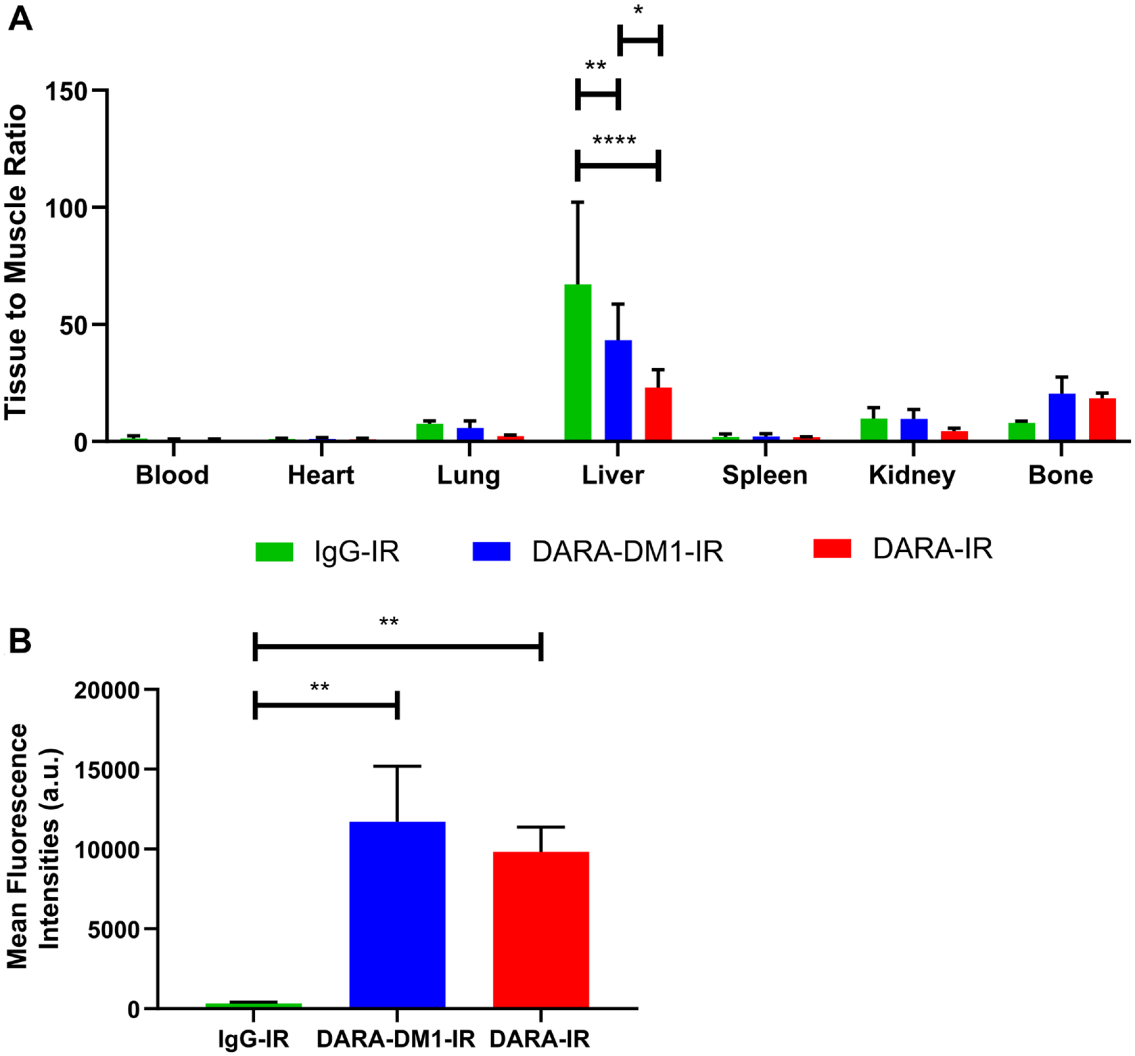
Biodistribution and flow cytometric analysis of DARA-DM1-IR in MM.1S IV mice. (**A**) Normalized biodistribution (defined as TMR) of DARA-DM1-IR, DARA-IR and IgG-IR 9 days after administration of fluorescent conjugate. *n* = 3–4/group. Two-way ANOVA followed by Sidak’s multiple comparisons test was performed on biodistribution data. (**B**) Flow cytometric analysis of IRDye800 MFIs from excised bone marrow of DARA-DM1-IR, DARA-IR and IgG-IR-injected mice. One-way ANOVA followed by Tukey’s multiple comparisons test was performed on flow cytometry data. *n* = 3–4/group. ^*^ *p* < 0.05; ^**^ *p* < 0.01; ^***^ *p* < 0.001; ^****^ *p* < 0.0001. Error bars represent standard deviation.

In vivo fluorescent imaging of DARA-DM1-IR was also performed in the MM.1S IV mouse model. Similar to the SQ mouse model, DARA-DM1-IR showed specificity to GFP^+^ tumors in marrow-rich regions such as the skull, long bones and spine (Figure 4A and 4B) 9 days post administration. Tissue biodistribution studies in tumor and non-tumor tissue from mice injected with DARA-DM1-IR and DARA-IR, respectively, showed similar uptake of both conjugates in bone regions (DARA-DM1-IR TMR: 20.4 ± 7.2; DARA-IR TMR: 18.4 ± 2.2) and greater uptake than in bones from mice injected with a non-specific IgG-IR (IgG-IR TMR: 8.2 ± 1.1). Liver uptake of DARA-DM1-IR (TMR: 43.3 ± 15.4) was similar to IgG-IR (TMR: 43.8 ± 6.6) and was higher than the uptake of DARA-IR (TMR: 23.0 ± 7.6) (Figure 5A). Flow cytometry on the excised bone marrow, measured as mean fluorescence intensities (MFI), confirmed *in vivo* and *ex vivo* images and showed significantly increased uptake of DARA-DM1-IR (MFI: 9832 ± 1545) and DARA-IR (MFI: 11715 ± 3475) relative to IgG-IR (MFI: 319 ± 86.75) with no significant difference between uptake of DARA-DM1-IR and DARA-IR (Figure 5B).

## DISCUSSION

CD38 is highly expressed on MM cells and is involved in their development and proliferation, making CD38 an attractive therapeutic target. DARA was the first Food and Drug Administration (FDA)-approved anti-CD38 immunotherapy prescribed to relapsed and refractory MM patients. While DARA is well tolerated and has robust clinical efficacy, not all heavily pretreated patients respond to single-agent DARA, necessitating other therapeutic agents to overcome this resistance [3, 11]. Several mechanisms have been proposed that are driving DARA resistance, including upregulation of complement inhibitors, clonal selectivity and microenvironmental interactions with bone marrow stromal cells [12]. CD38 expression is excluded as a possible reason, as responders and non-responders of DARA treatment show a marked reduction in CD38 [13]. A highly appealing strategy is to utilize the targeting power of antibodies as carriers of potent effector moieties to the target tumor cell. With the recent success of clinically-approved ADCs in MM, such as Blenrep (anti-BCMA) [14], and other ADCs undergoing clinical trial, such as IMGN901 (anti-CD56) [15], the potential of ADCs and similar immunoconjugates will be increasingly evaluated in such heavily pretreated MM patients. The general mechanism of cytotoxicity for such immunoconjugates includes binding and subsequent internalization into the cell, cleavage of the linker-drug complex and the release of the payload for killing the target cell [16]. The payloads of the ADCs described previously either damage DNA or impede microtubule assembly while retaining all the mechanisms of action of the native antibody, introducing an additional mechanism to overcome drug insensitivity [17, 18]. We reasoned that a similar conjugation of a cytotoxic drug to DARA could also widen its therapeutic window.

Here we demonstrate the use of DARA conjugated to the non-cleavable linker SMCC and the maytansinoid DM1 because of the improved *in vivo* stability and reduced bystander killing associated with drug payloads linked *via* non-cleavable linkers [4]. Free DM1 has also shown significant cytotoxicity to MM cells *in vitro* [19]. DARA has demonstrated preferential killing of high CD38-expressing MM cells, allowing for expansion of MM cells with low CD38 (CD38^low^) expression with reduced CDC and ADCC killing [13]. With the immunosuppressive nature of non-responders to DARA therapy [12, 20, 21], we believe the ADC could potentially target and kill CD38^low^ MM cells in the presence of reduced effector cell populations that DARA is reliant upon. We anticipate that DARA-DM1 will allow for a greater percentage of DM1 drug to reach tumors, lowering the minimum effective dose and elevating the maximum tolerated dose of drug payload. To evaluate the binding, internalization and distribution of DARA-DM1, we labelled the ADC with the NIR fluorophore IRDye800. With the advent of anti-CD38 ADCs [22], radiotherapies [23] as well as other classes of immunotoxins for use in MM [18], the NIR-labelling techniques applied in this manuscript can be used to visualize similar immunoconjugates in preclinical MM models *in vitro* and *in vivo* for more efficient clinical translation in MM.

At a DAR of 3.2, DARA-DM1 was found to have less than 10% DARA remain unconjugated. As expected with lysine-based conjugation, a heterogeneous distribution of 1–5 DM1 molecules was conjugated to DARA. While IRDye800 also requires free lysines for conjugation, preferentially on the heavy chain of the antibody [24, 25], absorbance and fluorescence spectroscopy demonstrated a similar DOL of ~1.3-1.4 between DARA-DM1-IR and DARA-IR without any differences in fluorescence emission. This suggests that the presence of DM1 and the heterogeneity of lysine conjugation did not inhibit labelling of IRDye800 to the DARA antibody.

Two human MM cell lines, MM.1S and U266, with different levels of CD38 expression were utilized to evaluate the cytotoxicity and mechanism of action of the DARA immunoconjugates. We demonstrated that conjugating DM1 enhanced cytotoxicity of the native DARA in a CD38-dependent fashion. DARA, conversely, did not demonstrate any cytotoxicity in either cell line, likely due to the lack of effector cells for the antibody to induce cell killing. Our flow cytometry and fluorescence microscopy studies showed that these differences in cytotoxicity may be due to differences in binding and internalization of DARA-DM1 in the MM.1S and U266 cells. While DARA-DM1 bound to both cell lines, we showed that there is significantly reduced binding and lysosomal internalization intracellularly in U266 cells than in MM.1S cells, corresponding with reduced CD38 expression and thus requiring greater concentration of DARA-DM1 to induce cytotoxicity. This is consistent with ADCs composed of non-cleavable linkers, which require lysosomal proteolytic degradation of the antibody [26]. These studies, additionally, corroborated that the conjugation of IRDye800 at a DOL of ~1.3–1.4 did not affect the stability or functional properties of DARA or DARA-DM1.

DARA-DM1 demonstrated significant *in vivo* single-dose efficacy within ~ 4 days in MM.1S IV mice compared to the native DARA. While DARA-treated mice showed reduced tumor burden 33 days post-tumor inoculation, DARA-DM1-treated mice showed early and near complete elimination of tumor burden with no weight loss observed in either mouse cohort. DARA monotherapy was likely efficacious *in vivo* due to ADCP activity [27] with the presence of normal macrophage and granulocyte populations in SCID beige mice [28]. These results demonstrated that, while DARA monotherapy still had significant therapeutic efficacy, conjugation of DM1 to DARA allowed for early and sustained elimination of tumor burden. DARA-DM1-IR and DARA-IR were imaged in MM.1S SQ and MM.1S IV mice to evaluate the tissue biodistribution and tumor targeting of DARA-DM1 in relation to DARA. Our *in vivo* imaging results showed that both immunoconjugates had high specificity to GFP^+^ tumor lesions in both mouse models. We, and others, have previously demonstrated that IV injection of human myeloma cells results in diffuse tumor growth in variable regions of bone marrow in mice compared to the localized tumor burden observed in MM.1S SQ mice [29, 30]. MM is a plasma cell disorder that causes significant skeletal morbidity within the bone marrow niche. As anticipated, both DARA-DM1-IR and DARA-IR uptake was observed in bone marrow regions in the MM.1S IV mice including skull, long bones and spine. Spatial tumor heterogeneity is characteristic of MM and the presence of circulating tumor cells may be contributing to the uptake of MM cells in different bone regions. Differences in the efficiency of tail vein injections of MM.1S cells and mouse-to-mouse variability in cytokines such as IL-6 may additionally be why some mice develop more tumor burden in one region than others. SCID beige mice also have several, normal lymphocyte populations, which may contribute to allogeneic tumor rejection from the host immune system, leading to variability in tumor uptake [28].

*Ex vivo* biodistribution demonstrated significant clearance through liver by day 9 in MM.1S SQ and IV mice. Due to their large size (~150 kDa), IgG antibodies such as DARA are primarily catabolized *via* the liver [31]. The greater hepatic uptake of DARA-DM1 relative to native DARA can be attributed to the higher hydrophobicity associated with ADCs, resulting in greater reticuloendothelial system clearance [32, 33]. These differences in liver uptake did not affect the tumor targeting ability of DARA-DM1, as seen in the *ex vivo* biodistribution as well as fluorescent microscopy and flow cytometry studies on the excised tumor tissue, but may have contributed to the differences in TBR at later time points when compared to DARA in the MM.1S SQ mice. It should be noted that DARA does not bind to murine CD38, therefore, in combination with the lack of a competent immune system, mice are not an ideal species for evaluating the off-target toxicity of the intact humanized ADCs. Future studies in humanized, immunocompetent mouse models of MM that can recapitulate the microenvironmental interactions with MM tumors are warranted.

Our studies demonstrate that conjugation of DM1 to the native DARA significantly enhanced its therapeutic efficacy *in vitro* and *in vivo*. Fluorophore labelling did not affect the stability or activity of the biologic and showed that both DARA-IR and DARA-DM1-IR had similar binding and biodistribution profiles. These imaging techniques can be applied to other immunotherapies and antibody conjugates under investigation in animal models of diverse hematologic cancers to evaluate similar parameters demonstrated in this article. Future studies can help in mechanistically understanding these therapies to enhance response and overcome resistance in treatment of these cancers.

## MATERIALS AND METHODS

### Maytansinoid and fluorophore conjugation of DARA

Daratumumab (Darzalex, Janssen) was generously donated by the Centre of Advanced Medicine pharmacy, Washington University in St. Louis. DARA-DM1 was synthesized through a one-step reaction. The non-cleavable linker, SMCC, conjugated to DM1 (MedKoo Biosciences) was conjugated to DARA *via* one-step N-hydroxysuccinimide ester reaction at an antibody concentration of 2.5 mg/mL in 1X PBS for 2 hours. A DAR of 20 to 1 was used for conjugation. Unconjugated drug was removed by desalting Zeba spin columns (Thermo Fisher). The final DAR of DARA-DM1 was calculated to be 3.2 *via* mass spectrometry. DARA, DARA-DM1 and non-specific IgG (Sigma Aldrich) were then conjugated to the NIR dye, IRDye800 (Li Cor Biosciences), according to the manufacturer’s instructions. Briefly, antibodies were reacted at an antibody concentration of 2.5 mg/mL in 0.1M potassium phosphate buffer (pH 8.5) for 2 hours. Dye to antibody molar ratio of 3 to 1 was used. Unconjugated dye was also removed by desalting Zeba spin columns. The DOL was determined using the DU-640B spectrophotometer (Beckman Coulter) to measure fluorophore absorbance at 774 nm and antibody absorbance at 280 nm, corrected for the fluorophore (Supplementary Figure 1). The DOL is defined as the average dye to protein concentration ratio.

Fluorescence emission was measured using the Fluorolog-3 spectrofluorometer (Horiba). After purification, conjugates were run on sodium dodecyl sulfate-polyacrylamide gel electrophoresis (SDS-PAGE) (Bio-Rad) in the presence of 1X PBS and human serum (Sigma Aldrich) at 37°C at incubation intervals of 1, 5 and 7 days. Gels were scanned using the Odyssey CLx (Li Cor) measured at 800 nm channel, and images were analyzed in Li Cor Image Studio version 5.2 software.

### Cell culture

The human myeloma MM.1S and U266 cells were obtained from ATCC. MM.1S cells were modified to express GFP and click beetle red luciferase (MM.1S-GFP-luc) by the DiPersio laboratory (Professor John F. DiPersio, Department of Medicine, Washington University School of Medicine, St Louis, USA) in 2014. Cells were tested negative for mycoplasma by the Washington University Genome Engineering and induced Pluripotent Stem Cell Core *via* MycoAlert PLUS Mycoplasma Detection Kit (Lonza) in 2014 and 2018. All cell lines were passaged 4–5 times following thaw before use in *in vitro* and *in vivo* studies. Cells were cultured in Roswell Park Memorial Institute (RPMI) 1640 medium (Thermo Fisher Scientific) supplemented with 10% heat inactivated fetal bovine serum (FBS) (Sigma Aldrich) and 1% penicillin/streptomycin (Inveon) at 37°C in a humidified environment with 5% CO_2_.

### Cytotoxicity studies

The *in vitro* activity of DARA conjugates was tested using the CellTiter 96 AQueous One Solution Cell Proliferation Assay (Promega) on MM.1S and U266 cells plated at 2.5 × 10^4^ cells per well in 96-well round-bottomed plates in triplicate and exposed to ADCs at different concentrations (0–10 μg/mL) for 72 hours. IC_50_ values for ADCs were calculated with GraphPad Prism Version 9.1.0 software.

### Cell uptake and internalization studies

MM.1S and U266 cells were incubated with 75 μg/mL of DARA-DM1-IR in 1X PBS for 1 hour. Cells were then washed twice in fluorescence activated cell sorting (FACS) buffer (made with 1X PBS, 0.5M Ethylenediaminetetraacetic acid disodium salt dihydrate (EDTA) (Corning) and 0.5% Bovine Serum Albumin (Inveon)). Non-specific binding was determined by incubating cells in the presence of excess (50-fold) unlabelled DARA for 1 hour before incubating with DARA-DM1-IR. Cells were immediately analyzed on the LSR Fortessa (BD).

To evaluate the lysosomal uptake of the DARA conjugates, MM.1S and U266 cells were incubated with 75 μg/mL of DARA-DM1-IR and DARA-IR, respectively, for 3 hours. Cells were washed twice in 1X PBS and fixed and permeabilized with CytoFAST Fix and Perm buffer (BioLegend). Cells were then stained with PE anti-human CD107a (LAMP-1) antibody (BioLegend) in the dark for 30 minutes. Cells were washed twice in FACS buffer and immediately analyzed on the LSR Fortessa. 7-aminoactinomycin D (Thermo Fisher Scientific) (7AAD)^–^ population was considered as viable tumor cells and used for statistical analysis for both studies. PE mouse anti-human CD38 (BD) was used to evaluate CD38 expression on MM.1S and U266 cells. Blue laser (Ex. 488 nm) was used to detect 7AAD (Em. 695/40 nm), yellow laser (Ex. 552 nm) was used to detect PE (Em. 585/15 nm) while red laser (Ex. 640 nm) was used to detect IRDye800 (Em. 780/60 nm). Flow cytometry data was analyzed with FlowJo Version 10.6.2 software.

### Live cell microscopy

300,000 MM.1S-GFP-luc cells/mL were seeded into 6-well tissue culture-treated plates (Corning) and incubated with 150 μg/mL of DARA-IR and DARA-DM1-IR at 37°C for 3 hours in 1X PBS. Cells were then washed twice in 1X PBS and immediately imaged on Cell Discoverer 7 (Zeiss) in the GFP (Ex./Em. 465 nm/520 nm) and Cy5 (Ex./Em. 640 nm/680 nm) wavelength channels with a 20X objective. Microscopy images were acquired with Zeiss ZEN 3.2 (blue edition) software and exported to and analyzed with NIH Image J software.

### Animal models

All animal studies were performed in accordance with the Institutional Animal and Use Committee of Washington University School of Medicine. Mice were anesthetized for all treatments and imaging with 2% v/v isoflurane/100% O_2_. Female 1–3 month old fox chase SCID mice (Charles Rivers Laboratories) were injected with 3 × 10^6^ MM.1S-GFP-luc cells in 100 μL 1X PBS SQ or IV *via* lateral tail vein. Tumor burden was monitored weekly *via* BLI prior to administration of DARA conjugates in both mouse models. For imaging studies in MM.1S SQ and IV mice, mice were randomized into respective cohorts when a mean bioluminescence flux of 1 × 10^9^ photons/second was achieved.

### *In vivo* therapy studies

Weekly BLI was performed on MM.1S IV mice until they reached a mean bioluminescence flux of 6 × 10^6^ photons/second. Mice were then randomized into untreated and treated cohorts (*n* = 6–7/group). Treated mice were provided DARA and DARA-DM1 intraperitoneally (i.p.) at a single dose of 4 mg/kg of body weight in 1X PBS. Additional BLI was performed twice per week to measure tumor cell viability for 6–7 weeks.

### *In vivo* and *ex vivo* fluorescence imaging studies

DARA-DM1-IR and DARA-IR, respectively, was administered IV in 1X PBS at 4 mg/kg of body weight in MM.1S SQ and MM.1S IV mice (*n* = 3–4/group). For MM.1S SQ mice, daily optical imaging in the GFP (Ex./Em. 480 nm/535 nm) and IRDye800 channels (Ex./Em. 785 nm/820 nm) was performed up to 9 days following injection using the IVIS Spectrum CT (Perkin Elmer). For MM.1S IV mice, optical imaging in the GFP (Ex./Em. 480 nm/535 nm) and IRDye800 channels (Ex./Em. 785 nm/820 nm) was performed 9 days following injection using the Optix MX3 time-domain diffuse optical imaging system (Advanced Research Technologies). Prior to imaging, hair was removed by gentle clipping and depilatory cream to improve light transmission. Following imaging, mice were sacrificed at appropriate time points and tissue was excised. GFP (Ex./Em. 480 nm/535 nm) and IRDye800 (Ex./Em. 780 nm/820 nm) fluorescent images of excised tissue were acquired on the IVIS Spectrum CT (Perkin Elmer), respectively. TBRs were calculated from IRDye800 fluorescent images of MM.1S SQ mice by drawing equivalently-sized ROIs in NIH ImageJ software around GFP^+^ tumor-bearing regions and non-fluorescent (background) regions on the contralateral side of the mouse and measuring total radiant efficiency (TRE). TMRs in *ex vivo* GFP and IRDye800 fluorescent images were calculated in Perkin Elmer Living Image 4.7.1 software. Analysis of *ex vivo* fluorescent images was performed by measuring TRE from ROIs drawn around bone and muscle tissue. TMRs were calculated by dividing TREs of tissue by TREs of muscle tissue of each respective mouse.

### Fluorescence immunohistochemistry

MM.1S SQ mice were euthanized 9 days post administration of DARA-IR and DARA-DM1-IR and tumors were resected, flash frozen in Tissue-Tek optimal cutting temperature (OCT) compound (Sakura) at –80°C and cut for histology on a cryostat (5-μm slices). Slices were stained with Hoechst 33342 (Fisher Scientific) and microscopy was performed using an upright Olympus BX51microscope (Olympus) equipped with a 40X objective and 405, 488 and 745 nm lasers. Tumor images were collected with Olympus Cells Standard 1.6 software and exported into NIH ImageJ software for analysis.

### *Ex vivo* flow cytometry

Viable cells were obtained from tibial and femoral bone marrow flush from MM.1S IV mice, washed in FACS buffer, stained and immediately analyzed with LSR Fortessa. For GFP^+^ tumor graft viability, 7AAD^–^/GFP^+^ population was considered as viable tumor cells and lasers were used as previously described. Blue laser (Ex. 488 nm) was used to detect FITC (Em. 530/30 nm). Binding of DARA-IR and DARA-DM1-IR to MM cells in the bone marrow was assessed using MFIs of IRDye800. Flow cytometry data was analyzed with FlowJo Version 10.6.2 software as previously described.

### Statistical analysis

All data is presented as mean ± standard deviation and statistical analysis was performed using GraphPad Prism Version 9.1.0 software. Statistical significance between cohorts was calculated using Student *t*-test and one-/two-way analysis of variance (ANOVA) followed by Sidak’s multiple comparisons test, unless specified otherwise. *P* values of less than 0.05 were considered statistically significant.

## SUPPLEMENTARY MATERIALS



## Abbreviations

DARA: Daratumumab
ADC: Antibody-drug conjugate
CD38: Cluster of Differentiation 38
SQ: Subcutaneous
IV: intravenous
IR: IRDye800
GFP: Green fluorescence protein
