# Correction: Tissue biodistribution and tumor targeting of near-infrared labelled anti-CD38 antibody-drug conjugate in preclinical multiple myeloma

**DOI:** 10.18632/oncotarget.28714

**Published:** 2025-04-11

**Authors:** Nicholas Cho, Sooah Ko, Monica Shokeen

**Affiliations:** ^1^Department of Radiology, Washington University School of Medicine, St. Louis, MO, USA; ^2^Department of Biomedical Engineering, Washington University in St. Louis, St. Louis, MO, USA; ^3^Alvin J. Siteman Cancer Center, Washington University School of Medicine and Barnes-Jewish Hospital, St. Louis, MO, USA

**This article has been corrected:** Oncotarget’s investigation of the paper is now complete. We’ve determined that in Figure 3A, the Day 0 representative image of MM.1S SQ mice in the “DARA-IR” group was unintentionally duplicated from the Day 2 image, taken before the fluorescent imaging. This error happened during the figure assembly process. The authors have removed the Day 0 images for both groups of mice. As stated in the Figure 3A caption, the figure is intended to show mice at days 2 and 9 post-dye administration. The authors declare that these modifications to the figures do not change the results or conclusions of the paper.

Original article: Oncotarget. 2021; 12:2039–2050. 2039-2050. https://doi.org/10.18632/oncotarget.28074

**Figure 3:**
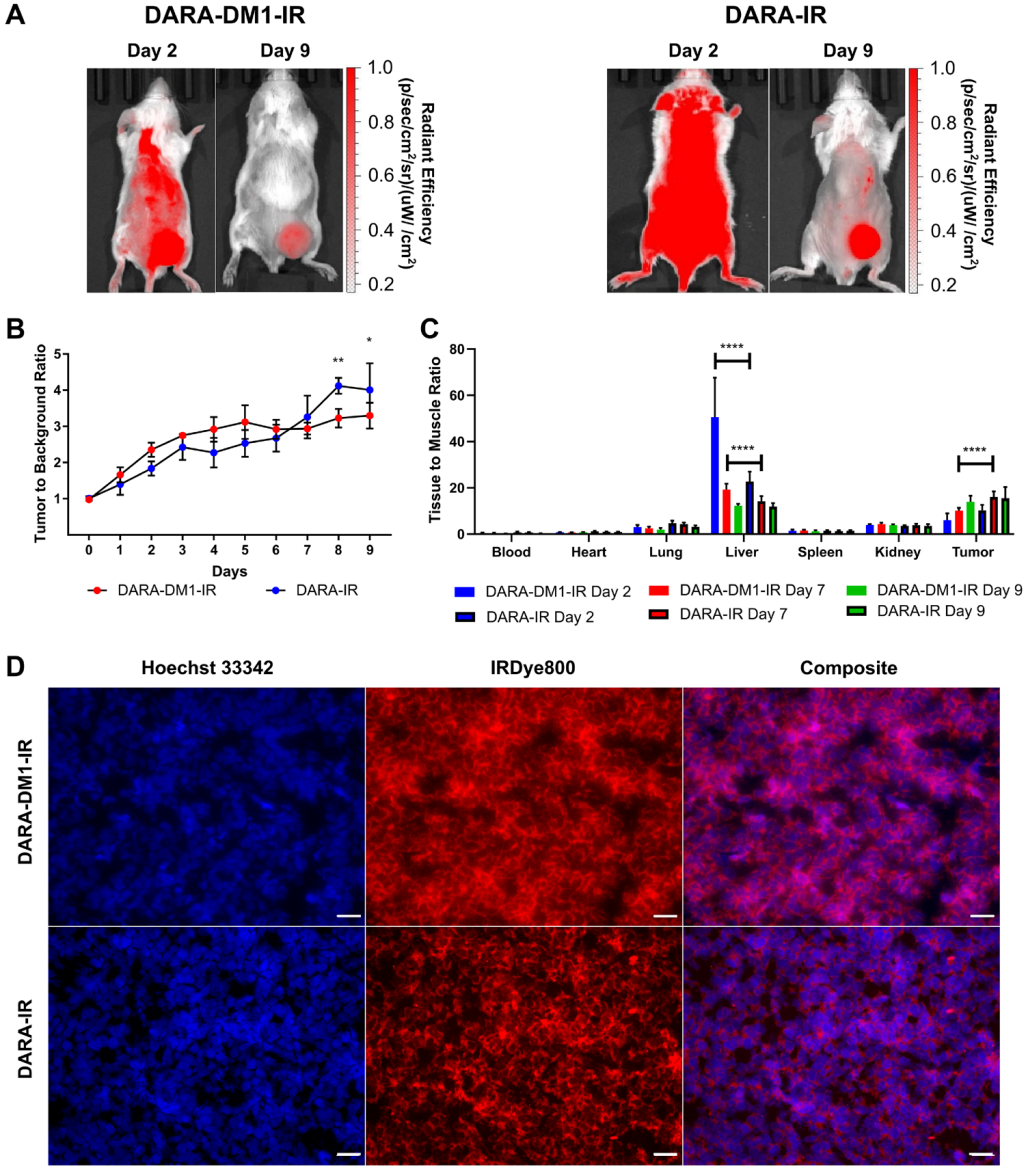
High contrast observed with DARA-DM1-IR at longer time points in MM.1S SQ mice. (**A**) Representative IRDye800 in vivo images of MM.1S SQ mice 2 and 9 days post administration of DARA-DM1-IR and DARA-IR. (**B**) Plot of calculated Tumor to Background Ratios (TBRs) in MM.1S SQ mice across individual time points following administration of DARA-DM1-IR and DARA-IR. Background is defined as the non-tumor, contralateral left flank of the mouse. Repeated measures two-way ANOVA followed by Sidak’s multiple comparison’s test was performed on TBR data. *n* = 3–4/group. (**C**) Normalized biodistribution (defined as tissue to muscle ratio (TMR)) of DARA-DM1-IR 2, 7 and 9 days after administration of fluorescent conjugate. *n* = 3–4/group. Two-way ANOVA followed by Sidak’s multiple comparisons test was performed on biodistribution data. (**D**) Immunohistochemistry of excised tumor sections from mice injected with DARA-DM1-IR and DARA-IR. Nuclear stain was performed with Hoechst 33342. Magnification: 40×; Scale bar: 100 μm. ^*^ *p* < 0.05; ^**^ *p* < 0.01; ^***^ *p* < 0.0001. Error bars represent standard deviation.

