# Supplementary material for: Tissue biodistribution and tumor targeting of near-infrared labelled anti-CD38 antibody-drug conjugate in preclinical multiple myeloma
Source: Oncotarget. 2021 Sep 28;12(20):2039–50. doi: 10.18632/oncotarget.28074 (PMC8487729; doi:10.18632/oncotarget.28074)
Supplement: Supplementary file 1 [file oncotarget-12-2039-s001.pdf]

# Tissue biodistribution and tumor targeting of near-infrared labelled anti-CD38 antibody-drug conjugate in preclinical multiple myeloma

## SUPPLEMENTARY MATERIALS

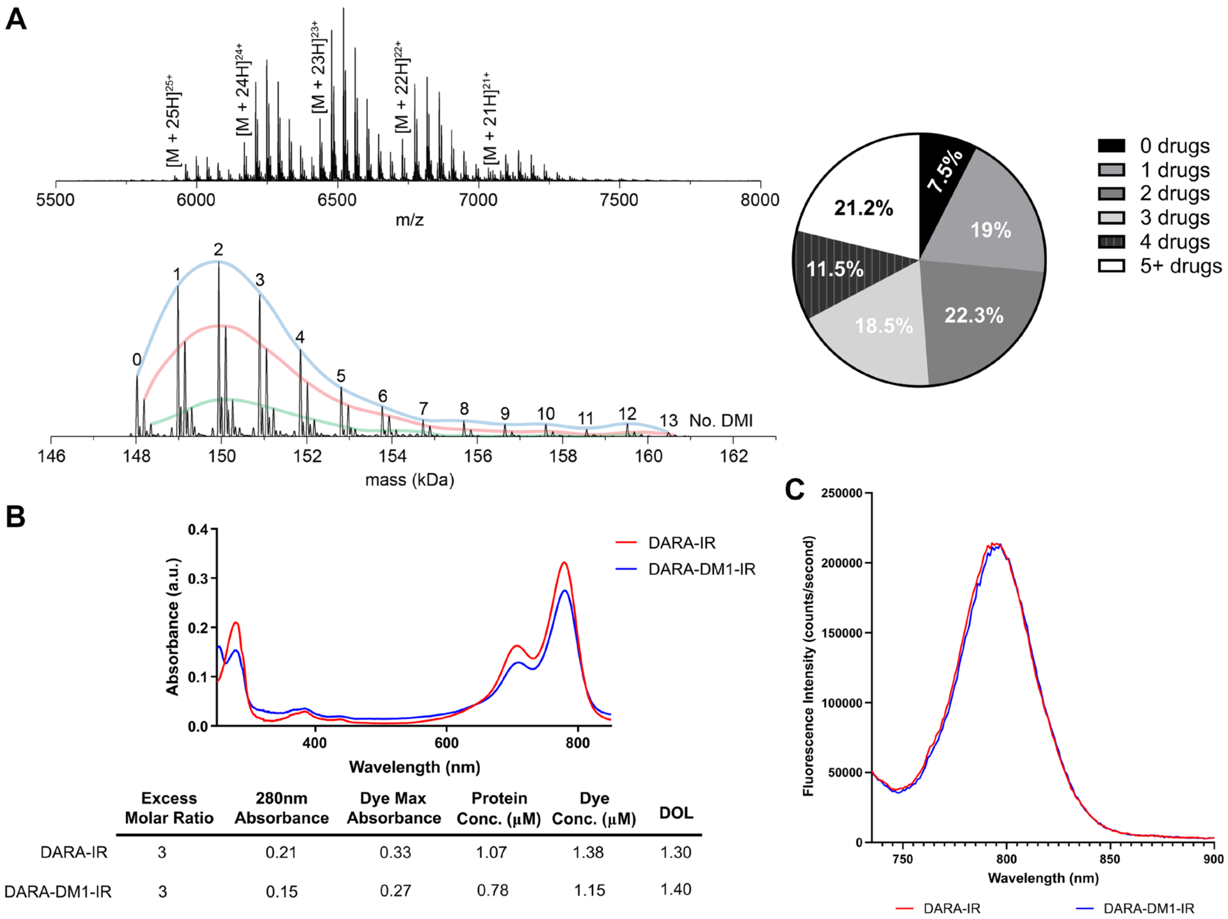


| | Excess Molar Ratio | 280nm Absorbance | Dye Max Absorbance | Protein Conc. (µM) | Dye Conc. (µM) | DOL |
|---|---|---|---|---|---|---|
| DARA-IR | 3 | 0.21 | 0.33 | 1.07 | 1.38 | 1.30 |
| DARA-DM1-IR | 3 | 0.15 | 0.27 | 0.78 | 1.15 | 1.40 |

**Supplementary Figure 1: Analytical characterization of DARA-DM1 and DARA-DM1-IR.** (**A**) Representative mass spectrometry spectra and percent distribution of DARA with 1, 2, 3, 4 or 5+ DM1 drugs per antibody at a 20 to 1 conjugation ratio. (**B**) Antibody-dye absorbance spectra of DARA-DM1-IR and DARA-IR. After reaction and purification, the absorbance spectrum for DARA-DM1-IR and DARA-IR were used to determine the DoL as described in the methods section. DoL was determined by dividing bulk fluorophore concentration by antibody concentration. (**C**) Fluorescence emission spectra of DARA-DM1-IR and DARA-IR.

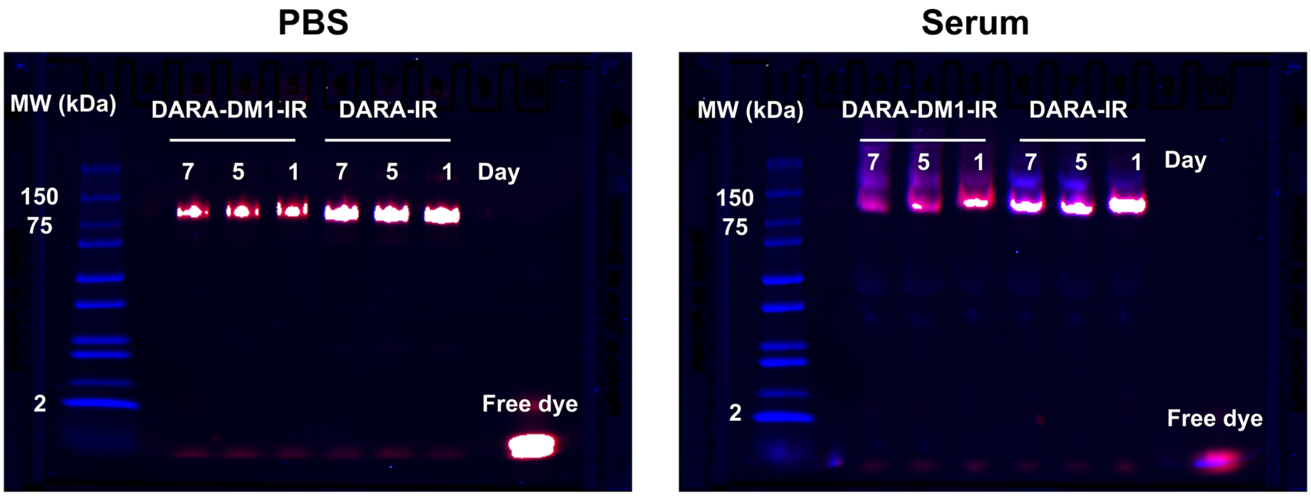


**Supplementary Figure 2: *In vitro* stability of DARA-DM1-IR and DARA-IR.** SDS-PAGE of DARA-DM1-IR and DARA-IR incubated in PBS and human serum between 1 and 7 days.

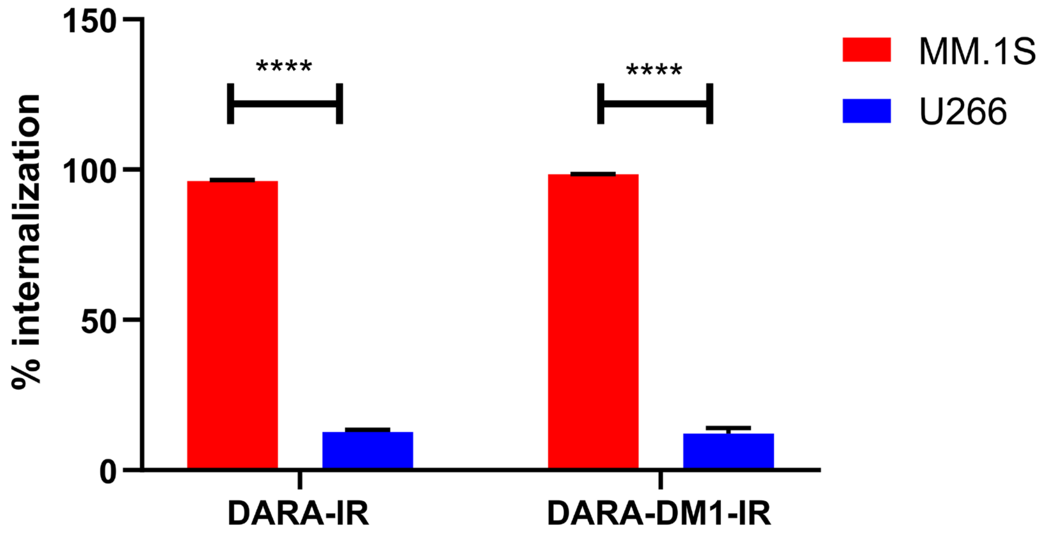


**Supplementary Figure 3: Internalization of DARA conjugates.** Flow cytometric analysis of percent of MM.1S and U266 cells internalized with DARA-IR and DARA-DM1-IR in LAMP-1$^{+}$ lysosomes 3 hours post-incubation. Student t-tests were performed for each conjugate and cell line. $^{****}p < 0.0001$. Error bars represent standard deviation.

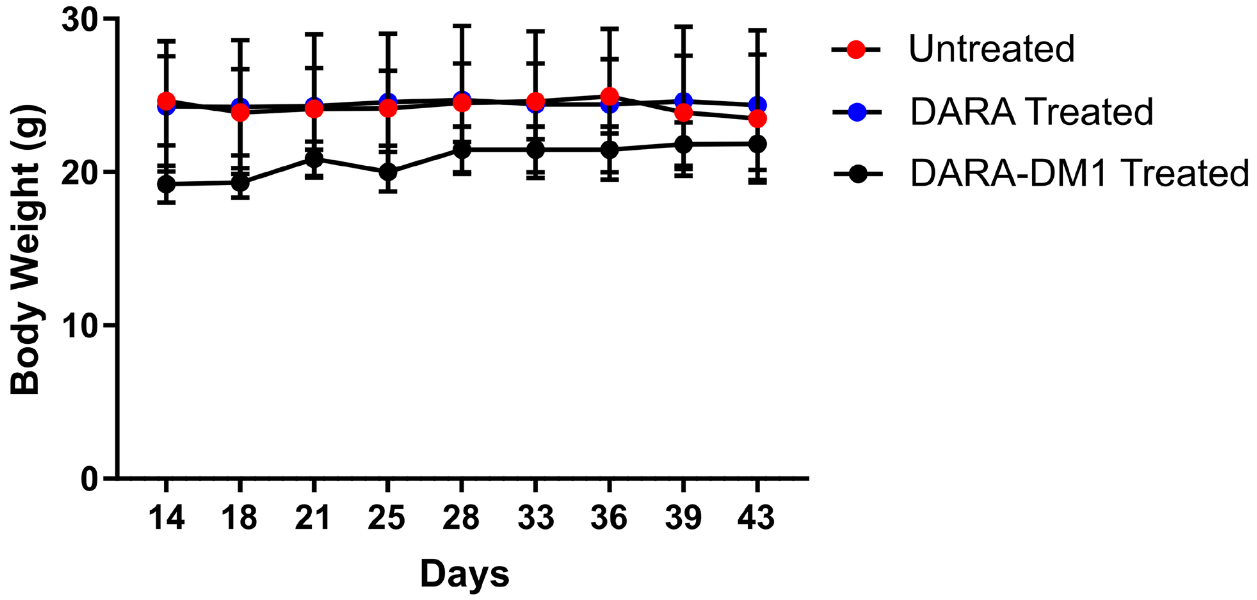


**Supplementary Figure 4: Body weights of untreated and treated MM.1S IV mice.** Mean body weights of MM.1S IV mice following treatment with DARA or DARA-DM1. Error bars represent standard deviation.
